# Intra-specific genetic variability dataset on *rbcL* gene in *Moringa oleifera* Lam. (Moringaceae) in Nigeria

**DOI:** 10.1016/j.dib.2023.109399

**Published:** 2023-07-11

**Authors:** Conrad Asotie Omonhinmin, Enameguono Ethel Olomukoro, Chinedu Charles Onuselogu, Jacob Olagbenro Popoola, Stephen Oluwanifise Oyejide

**Affiliations:** a Department of Biological Sciences/Biotechnology Cluster, College of Science and Technology, Covenant University, Ogun State, Nigeria; b Pure and Applied Biology Programme, Department of Biological Sciences, College of Agriculture, Engineering and Science, Bowen University, PMB 284, 232101, Iwo, Osun State, Nigeria; c Department of Cell Biology and Genetics, Faculty of Science, University of Lagos, Akoka, Nigeria

**Keywords:** *Moringa oleifera*, Conservation, Climate change, Drumstick, Genetic diversity, Medicinal, Phylogeny

## Abstract

*Moringa oleifera* is an underutilized perennial tree and is widely cultivated and studied due to its multi-purpose uses. Currently, not much information exists about the *rbcL* (Ribulose-1,5-bisphosphate carboxylase/oxygenase large subunit) gene sequence in *M. oleifera.* The *rbcL* gene is a conserved chloroplast gene and hence can be used for phylogenetic studies of plants at higher and lower taxonomic levels. The data consists of twenty-two *rbcL* gene sequences, nucleotide, and amino acid compositions of *M. oleifera* collected from sixteen states across Nigeria. The data offers an understanding of the genetic diversity and evolution of the species, which are crucial for both scientists and breeders working on the crop as well as for attempts to conserve the species.

Specifications Table

**Table:** 

Subject	Biological Sciences
Specific subject area	Agricultural, Genetic diversity, Phylogenetics, Evolution.
Type of data	Tables, Figures.
How data were acquired	PCR amplification and DNA Sanger sequencing for rbcL gene amplification.
Data format	Raw, Analyzed.
Description of data collection	In different parts of Nigeria (Abia, Anambra, Benue, Edo, Enugu, Gombe, Imo, Kano, Kwara, Niger, Ogun, Ondo, Osun, Oyo, Plateau, and Sokoto), young leaf samples of *M. oleifera* were collected. Table 1. The population diversity, nucleotide, and amino acid contents of each accession were determined using DnaSP 6.0, and all accessions were assessed using *rbcL* primers. CodonW was used to estimate the codon use indices.
Data source location	The data locations are summarized in Table 1.
Data accessibility	The sequence data of the accessions have been deposited in NCBI GenBank data base sequence and has the following accession numbers; MH243073.1, MH243074.1, MH243075.1, MH243076.1, MH243077.1, MH243078.1, MH243079.1, MH243080.1, MH243081.1, MH243082.1, MH243083.1, MH243084.1, MH243085.1, MH243086.1, MH243087.1, MH243088.1, MH243089.1, MH243090.1, MH243091.1, MH243092.1, MH243093.1, MH243094.1. https://www.ncbi.nlm.nih.gov/nuccore/?term=Moringa+oleifera+Omonhinmin

## 1 Value of the Data

• The data provides information on the distribution and genetic diversity of *M. oleifera* across the various ecological belts of Nigeria using information from partial rbcL gene sequences, nucleotide polymorphism and amino acid composition.
• The data offers genetic insights into the diversity and evolution of the species valuable to its conservation, breeding and genetic improvement.
• The rbcL gene sequences, amino acid composition and codon bias usage information are useful for molecular phylogeny, evolution, sub-speciation and ecological adaptability of the species.

## 2 Objective

To explore the chloroplast gene Ribulose-1,5-bisphosphate carboxylase/oxygenase large subunit - *rbcL* via sanger sequencing and generate data relevant for intra-specific genetic diversity, phylogenetic relationship, and ecological adaptability of *M. oleifera* in Nigeria.

## 3 Data Description

**Fig. 1:**
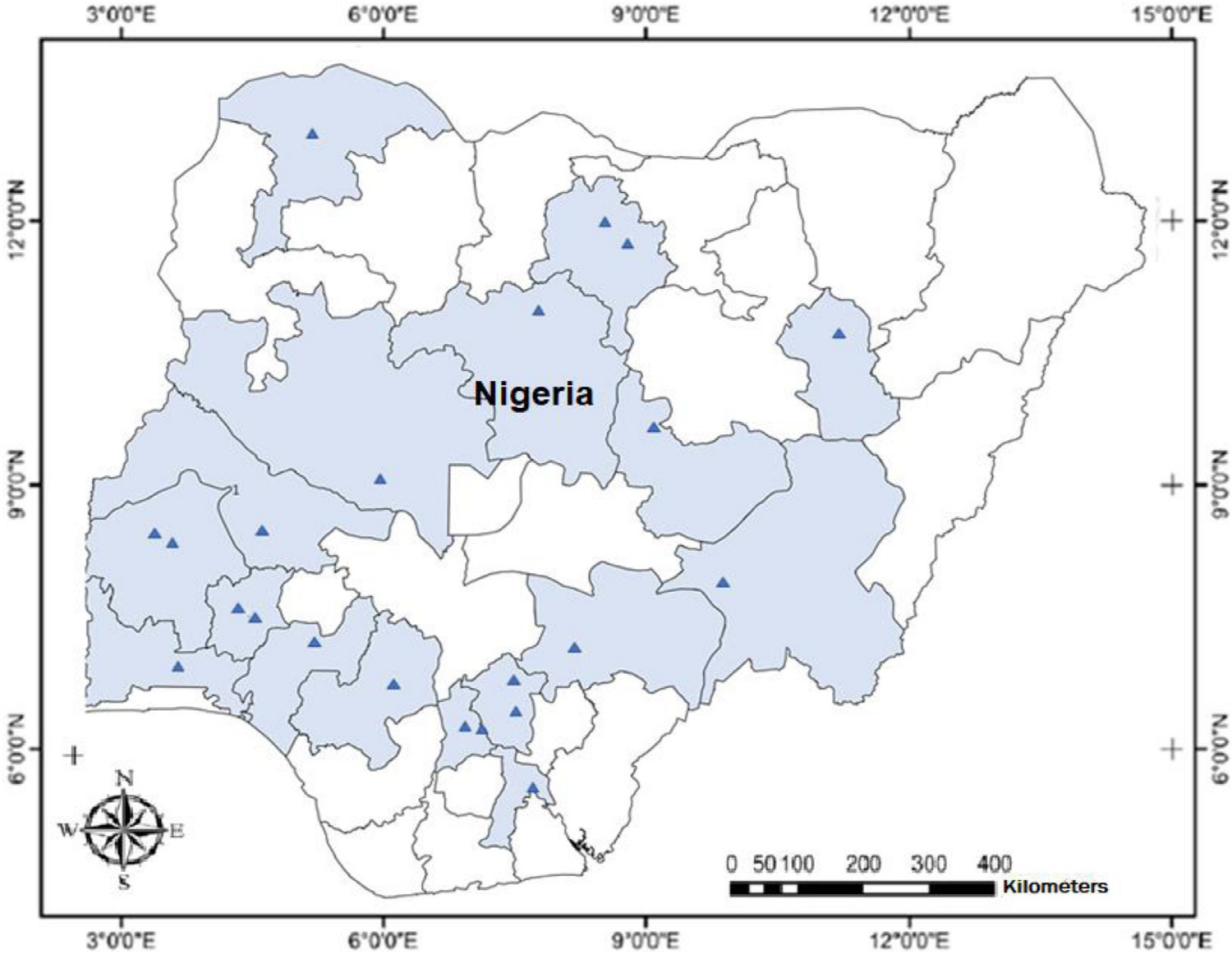
Collection range for *Moringa oleifera* accessions across Nigeria.

**Fig. 2:**
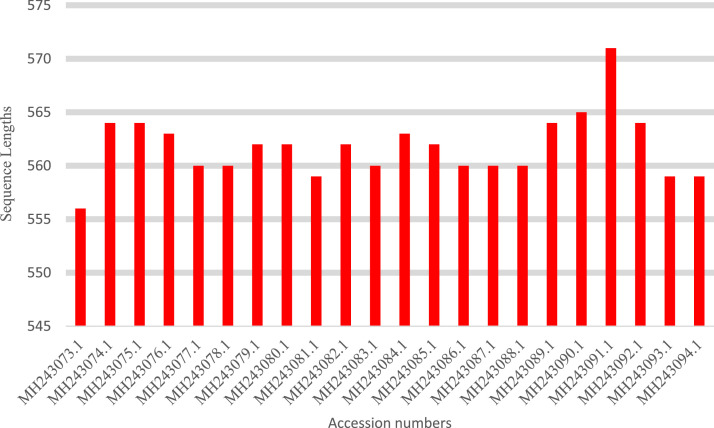
The *rbcL* Sequence Lengths Analysis for the 22 *M. oleifera* Accessions.

**Fig. 3:**
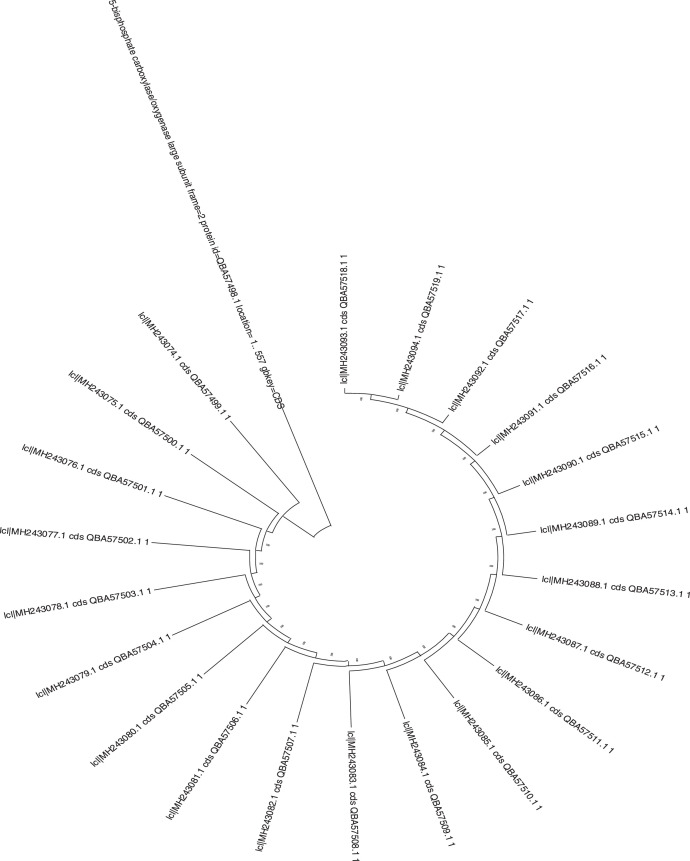
Phylogenetic Tree Construct of the *M. oleifera* rbcL Gene Sequences.

Analysis of *Moringa oleifera* accessions collected across Nigeria via sanger sequencing generated data relevant for intra-specific genetic diversity, phylogenetic relationship and ecological adaptability of the species. The rbcL gene is reported to be approximately 1400 bp in full length. The rbcL sequenced for the study recorded approximately 563 bp in length. Table 1; list the collection site information and NCBI Genbank details for the twenty-two *M. oleifera* accessions. Table 2; presents the sequence length and nucleotide content for the 22 accession sequences. Table 3; presents the intra-specific genetic diversity details of the 22 accessions based on sequences information. Table 4; shows the amino acid composition of the accession sequences. Table 5; represents the codon usage profile based on the 22 *M. oleifera* sequences. Table 6; highlights the Codon usage indices of each accession sequence. Fig. 1; shows the collections range of *M. oleifera* across Nigeria. Fig. 2; is the graphical representation of the sequence lengths of the 22 *M. oleifera* sequences, and Fig. 3; is the Phylogenetic tree construct using the 22 *M. oleifera* sequences.

**Table 1:** Collection Details, Passport data, and GenBank Accession Number of the *M. oleifera* Accessions for the Study.

SN	Herbarium Number (vouchers)	GenBank Accession Number	Area of collection	State	Latitude (NS)	Longitude (EW)
1	ABN057	MH243073.1	Okpanku	Abia	5.96114	7.66185
2	ANN046	MH243074.1	Unizik	Anambra	6.24884	7.11168
3	ANN049	MH243075.1	Enu Ifite	Anambra	6.24786	7.08769
4	BEN081	MH243076.1	Otukpo	Benue	7.19793	8.13467
5	EDN035	MH243077.1	Ehanlen-Ewu	Edo	6.73394	6.17659
6	ENN053	MH243078.1	Orba1	Enugu	6.84548	7.40968
7	ENN055	MH243079.1	Ugwuomu	Enugu	6.44541	7.53084
8	GON068	MH243080.1	Nafada	Gombe	11.09291	11.3334
9	KAN033	MH243081.1	Tudu Wada	Kaduna	11.0772	7.71346
10	KNN077	MH243082.1	Army Barracks	Kano	12.03227	8.5102
11	KNN078	MH243083.1	Kofar Kudu	Kano	11.95671	8.5273
12	KWN015	MH243084.1	Ilorin	Kwara	8.49105	4.54542
13	NIN018	MH243085.1	Bida	Niger	9.07459	6.00567
14	OGN026	MH243086.1	Olodo	Ogun	7.81441	4.34568
15	ONN070	MH243087.1	Owena Road	Ondo	7.28619	5.22675
16	OSN019	MH243088.1	Ipetumodu	Osun	7.53514	4.40535
17	OSN020	MH243089.1	OAU	Osun	7.50715	4.52791
18	OYN010	MH243090.1	Okaka	Oyo	8.45743	3.40868
19	PLN030	MH243091.1	Unijos	Plateau	9.95093	8.88948
20	SON066	MH243092.1	Sokoto	Sokoto	13.05757	5.24589
21	TAN085	MH243093.1	Wukari Road	Taraba	7.89721	9.80782
22	OYN001	MH243094.1	Oke-Oro / Saki	Oyo	8.66834	3.40054

**Table 2:** Sequence Length and Percentage Nucleotide Content of the rbcL Sequences for 22 *M. oleifera* Accessions.

SN	Accession Number	State	%GC	%A	%C	%G	%T	Sequence Length
1	MH243073.1	Abia	43.88	27.2	21.6	22.3	29.0	556
2	MH243074.1	Anambra	43.79	27.5	21.5	22.3	28.7	564
3	MH243075.1	Anambra	43.79	27.5	21.5	22.3	28.7	564
4	MH243076.1	Benue	43.87	27.5	21.5	22.4	28.6	563
5	MH243077.1	Edo	44.11	27.1	21.6	22.5	28.8	560
6	MH243078.1	Enugu	44.11	27.1	21.6	22.5	28.8	560
7	MH243079.1	Enugu	43.77	27.6	21.5	22.2	28.6	562
8	MH243080.1	Gombe	43.77	27.6	21.5	22.2	28.6	562
9	MH243081.1	Kaduna	44.01	27.2	21.6	22.4	28.8	559
10	MH243082.1	Kano	43.77	27.6	21.5	22.2	28.6	562
11	MH243083.1	Kano	44.11	27.1	21.6	22.5	28.8	560
12	MH243084.1	Kwara	43.87	27.5	21.5	22.4	28.6	563
13	MH243085.1	Niger	43.77	27.6	21.5	22.2	28.6	562
14	MH243086.1	Ogun	44.11	27.1	21.6	22.5	28.8	560
15	MH243087.1	Ondo	44.11	27.1	21.6	22.5	28.8	560
16	MH243088.1	Osun	44.11	27.1	21.6	22.5	28.8	560
17	MH243089.1	Osun	43.79	27.5	21.5	22.3	28.7	564
18	MH243090.1	Oyo	43.89	27.4	21.4	22.5	28.7	565
19	MH243091.1	Plateau	43.96	27.5	21.4	22.6	28.5	571
20	MH243092.1	Sokoto	43.79	27.5	21.5	22.3	28.7	564
21	MH243093.1	Taraba	44.01	27.2	21.6	22.4	28.8	559
22	MH243094.1	Oyo	44.01	27.2	21.6	22.4	28.8	559

**Table 3:** Genetic diversity and differentiation parameters (Mean and SE over Loci and Pops) for rbcL sequences of the 22 *M. oleifera* Accessions.

Parameter	N	Na	Ne	I	h	uh	PPL
Mean	7.327	2.642	2.166	0.810	0.499	0.584	96.86%
SE	0.041	0.017	0.014	0.007	0.004	0.004	2.08%
No. Loci	562						
No. Samples	22						

Sample Size (N), No. Alleles (Na), No. Effective Alleles (Ne), Information Index (i), Diversity (h) and Unbiased Diversity by Population (uh), Percentage of Polymorphic Loci (PPL).

**Table 4:** Amino Acid Composition of *M. oleifera* rbcL Sequences.

Amino acids	MH.73.1	MH.74.1	MH.75.1	MH.76.1	MH.77.1	MH.78.1	MH.79.1	MH.80.1	MH.81.1	MH.82.1	MH.83.1	MH.84.1	MH.85.1	MH.86.1	MH.87.1	MH.88.1	MH.89.1	MH.90.1	MH.91.1	MH.92.1	MH.93.1	MH.94.1
Ala	27.158	27.482	27.482	27.531	27.143	27.143	27.580	27.580	27.191	27.580	27.143	27.531	27.580	27.143	27.143	27.143	27.482	27.434	27.496	27.482	27.191	27.191
Cys	21.583	21.454	21.454	21.492	21.607	21.607	21.530	21.530	21.646	21.530	21.607	21.492	21.530	21.607	21.607	21.607	21.454	21.416	21.366	21.454	21.646	21.646
Asp	0.000	0.000	0.000	0.000	0.000	0.000	0.000	0.000	0.000	0.000	0.000	0.000	0.000	0.000	0.000	0.000	0.000	0.000	0.000	0.000	0.000	0.000
Glu	0.000	0.000	0.000	0.000	0.000	0.000	0.000	0.000	0.000	0.000	0.000	0.000	0.000	0.000	0.000	0.000	0.000	0.000	0.000	0.000	0.000	0.000
Phe	0.000	0.000	0.000	0.000	0.000	0.000	0.000	0.000	0.000	0.000	0.000	0.000	0.000	0.000	0.000	0.000	0.000	0.000	0.000	0.000	0.000	0.000
Gly	22.302	22.340	22.340	22.380	22.500	22.500	22.242	22.242	22.361	22.242	22.500	22.380	22.242	22.500	22.500	22.500	22.340	22.478	22.592	22.340	22.361	22.361
His	0.000	0.000	0.000	0.000	0.000	0.000	0.000	0.000	0.000	0.000	0.000	0.000	0.000	0.000	0.000	0.000	0.000	0.000	0.000	0.000	0.000	0.000
Ile	0.000	0.000	0.000	0.000	0.000	0.000	0.000	0.000	0.000	0.000	0.000	0.000	0.000	0.000	0.000	0.000	0.000	0.000	0.000	0.000	0.000	0.000
Lys	0.000	0.000	0.000	0.000	0.000	0.000	0.000	0.000	0.000	0.000	0.000	0.000	0.000	0.000	0.000	0.000	0.000	0.000	0.000	0.000	0.000	0.000
Leu	0.000	0.000	0.000	0.000	0.000	0.000	0.000	0.000	0.000	0.000	0.000	0.000	0.000	0.000	0.000	0.000	0.000	0.000	0.000	0.000	0.000	0.000
Met	0.000	0.000	0.000	0.000	0.000	0.000	0.000	0.000	0.000	0.000	0.000	0.000	0.000	0.000	0.000	0.000	0.000	0.000	0.000	0.000	0.000	0.000
Asn	0.000	0.000	0.000	0.000	0.000	0.000	0.000	0.000	0.000	0.000	0.000	0.000	0.000	0.000	0.000	0.000	0.000	0.000	0.000	0.000	0.000	0.000
Pro	0.000	0.000	0.000	0.000	0.000	0.000	0.000	0.000	0.000	0.000	0.000	0.000	0.000	0.000	0.000	0.000	0.000	0.000	0.000	0.000	0.000	0.000
Gln	0.000	0.000	0.000	0.000	0.000	0.000	0.000	0.000	0.000	0.000	0.000	0.000	0.000	0.000	0.000	0.000	0.000	0.000	0.000	0.000	0.000	0.000
Arg	0.000	0.000	0.000	0.000	0.000	0.000	0.000	0.000	0.000	0.000	0.000	0.000	0.000	0.000	0.000	0.000	0.000	0.000	0.000	0.000	0.000	0.000
Ser	0.000	0.000	0.000	0.000	0.000	0.000	0.000	0.000	0.000	0.000	0.000	0.000	0.000	0.000	0.000	0.000	0.000	0.000	0.000	0.000	0.000	0.000
Thr	28.957	28.723	28.723	28.597	28.750	28.750	28.648	28.648	28.801	28.648	28.750	28.597	28.648	28.750	28.750	28.750	28.723	28.673	28.546	28.723	28.801	28.801
Val	0.000	0.000	0.000	0.000	0.000	0.000	0.000	0.000	0.000	0.000	0.000	0.000	0.000	0.000	0.000	0.000	0.000	0.000	0.000	0.000	0.000	0.000
Trp	0.000	0.000	0.000	0.000	0.000	0.000	0.000	0.000	0.000	0.000	0.000	0.000	0.000	0.000	0.000	0.000	0.000	0.000	0.000	0.000	0.000	0.000
Tyr	0.000	0.000	0.000	0.000	0.000	0.000	0.000	0.000	0.000	0.000	0.000	0.000	0.000	0.000	0.000	0.000	0.000	0.000	0.000	0.000	0.000	0.000

**Table 5:** Codon Usage of *M. oleifera* rbcL Sequences.

Codon	Count	RSCU	Codon	Count	RSCU	Codon	Count	RSCU	Codon	Count	RSCU
UUU(F)	4	1.00	UCU(S)	5	3.75	UAU(Y)	8	1.33	UGU(C)	2	1.33
UUC(F)	4	1.00	UCC(S)	1	0.75	UAC(Y)	4	0.67	UGC(C)	1	0.67
UUA(L)	2	1.13	UCA(S)	0	0.00	UAA(*)	0	0.00	UGA(*)	0	0.00
UUG(L)	4	1.50	UCG(S)	0	0.00	UAG(*)	0	0.00	UGG(W)	2	1.00
CUU(L)	3	1.13	CCU(P)	7	2.15	CAU(H)	1	1	CGU(R)	3	1.80
CUC(L)	0	0.00	CCC(P)	4	1.23	CAC(H)	1	1	CGC(R)	2	1.20
CUA(L)	4	1.50	CCA(P)	2	0.62	CAA(Q)	3	1.5	CGA(R)	3	1.80
CUG(L)	2	0.75	CCG(P)	0	0.00	CAG(Q)	1	0.5	CGG(R)	0	0.00
AUU(I)	4	1.50	ACU(T)	10	2.67	AAU(N)	2	0.8	AGU(S)	1	0.75
AUC(I)	4	1.50	ACC(T)	3	0.80	AAC(N)	3	1.2	AGC(S)	1	0.75
AUA(I)	0	0.00	ACA(T)	2	0.53	AAA(K)	10	1.67	AGA(R)	2	1.20
AUG(M)	1	1.00	ACG(T)	0	0.00	AAG(K)	2	0.33	AGG(R)	0	0.00
GUU(V)	6	2.00	GCU(A)	8	2.00	GAU(D)	7	1.56	GGU(G)	7	1.65
GUC(V)	0	0.00	GCC(A)	3	0.75	GAC(D)	2	0.44	GGC(G)	1	0.24
GUA(V)	4	1.33	GCA(A)	2	0.50	GAA(E)	9	1.50	GGA(G)	6	1.41
GUG(V)	2	0.67	GCG(A)	3	0.75	GAG(E)	3	0.50	GGG(G)	3	0.71

⁎ RSCU: Relatively synonymous codon usage.

**Table 6:** Codon Usage Indices per Accession for the 22 *M. oleifera* rbcL Sequences.

Accessions	Codon Usage Parameters												
		T3s	C3s	A3s	G3s	CAI	CBI	Fop	Nc	GC3s	GC	L_sym	L_aa
MH243073.1		0.5065	0.2208	0.3497	0.1481	0.264	0.138	0.500	49.37	0.297	0.438	182	185
MH243074.1		0.5064	0.2179	0.3562	0.1449	0.269	0.141	0.503	49.33	0.292	0.438	185	188
MH243075.1		0.5064	0.2179	0.3562	0.1449	0.269	0.141	0.503	49.33	0.292	0.438	185	188
MH243076.1		0.5032	0.2194	0.3586	0.1460	0.268	0.137	0.500	49.44	0.293	0.437	184	187
MH243077.1		0.5032	0.2194	0.3542	0.1471	0.266	0.133	0.497	49.53	0.295	0.439	183	186
MH243078.1		0.5032	0.2194	0.3586	0.1460	0.268	0.137	0.500	49.44	0.293	0.437	184	187
MH243079.1		0.5032	0.2194	0.3586	0.1460	0.268	0.137	0.500	49.44	0.293	0.437	184	187
MH243080.1		0.5032	0.2194	0.3586	0.1460	0.268	0.137	0.500	49.44	0.293	0.437	184	187
MH243081.1		0.5032	0.2194	0.3542	0.1471	0.266	0.133	0.497	49.53	0.295	0.439	183	186
MH243082.1		0.5032	0.2194	0.3586	0.1460	0.268	0.137	0.500	49.44	0.293	0.437	184	187
MH243083.1		0.5032	0.2194	0.3542	0.1471	0.266	0.133	0.497	49.53	0.295	0.439	183	186
MH243084.1		0.5032	0.2194	0.3586	0.1460	0.268	0.137	0.500	49.44	0.293	0.437	184	187
MH243085.1		0.5032	0.2194	0.3586	0.1460	0.268	0.137	0.500	49.44	0.293	0.437	184	187
MH243086.1		0.5032	0.2194	0.3542	0.1471	0.266	0.133	0.497	49.53	0.295	0.439	183	186
MH243087.1		0.5032	0.2194	0.3542	0.1471	0.266	0.133	0.497	49.53	0.295	0.439	183	186
MH243088.1		0.5032	0.2194	0.3542	0.1471	0.266	0.133	0.497	49.53	0.295	0.439	183	186
MH243089.1		0.5064	0.2179	0.3562	0.1449	0.269	0.141	0.503	49.33	0.292	0.438	185	188
MH243090.1		0.5064	0.2179	0.3562	0.1449	0.269	0.141	0.503	49.33	0.292	0.438	185	188
MH243091.1		0.5096	0.2166	0.3514	0.1500	0.271	0.140	0.503	49.35	0.294	0.439	187	190
MH243092.1		0.5064	0.2179	0.3562	0.1449	0.269	0.141	0.503	49.33	0.292	0.438	185	188
MH243093.1		0.5032	0.2194	0.3542	0.1471	0.266	0.133	0.497	49.53	0.295	0.439	183	186
MH243094.1		0.5032	0.2194	0.3542	0.1471	0.266	0.133	0.497	49.53	0.295	0.439	183	186

## 4 Experimental Design, Materials and Methods

### 4.1 Plant material

Twenty-two (22) *Moringa oleifera* accessions were collected from 16 states in Nigeria across the four major agroecological zones traversing the country's land range [[1], [2], [3], [4]] and as stated in Table 1. The Silica-gel dried leaves were used for molecular analysis at the Bioscience Laboratory, International Institute of Tropical Agriculture (IITA), Ibadan Nigeria.

### 4.2 Genomic DNA extraction

Genomic DNA was extracted using the CTAB protocol as identified by [5].

### 4.3 DNA sequencing and gene amplification

The ribulose 1,5 bisphosphate carboxylase/oxygenase large subunit (*rbcL*) gene which served as a reference, was used to design the forward and reverse primers. The sequence of the forward primer (H1f) is: 5-CCACAAACAGAGACTAAAGC-3 (T_m_ = 51.1°C) and the reverse primer (Fofana R): 5-GTAAAATCAAGTCCACCGCG-3 (T_m_ = 54.2°C) [6]. The PCR amplicon was sequenced at the International Institute of Tropical Agriculture (IITA) in Ibadan, Nigeria using the ABI 3130x genetic analyzer (Applied Biosystems).

### 4.4 Data analysis

The Sanger sequences generated were aligned using ClustalW on BioEdit (ver. 7.2.5) with default settings to create consensus sequences [7]. Sequences were submitted on NCBI GenBank with accession numbers in Table 1. Population diversity indices such as numbers of segregating sites (S), haplotype number (h), haplotype diversity (Hd), and nucleotide diversity (π) were estimated using DNAsp 6.0 [8]. The nucleotide composition was estimated using Unipro UGENE v44.0 [9] and GenAlEx 6.5 [10]. Amino acid compositions and the codon usage frequency table of *M. oleifera* were estimated using DnaSP 6.0. Phylogenetic analyses were conducted in MEGA v11.0.13 using the neighbor-joining tree [11]. Codon usage indices were calculated using CodonW as implemented on a public Galaxy server (https://galaxy.pasteur.fr/).

## Ethics Statements

The field data presented in Table 1, were obtained via open field collection visits and did not require informed consent. No part of the data was obtained from any Social Media platform.

## CRediT Author Statement

**Conrad Asotie Omonhinmin:** Conceptualization, Methodology, Supervision, review manuscript; **Enameguono Ethel Olomukoro:** Experimentation, writing manuscript, submission of sequences on GenBank; **Chinedu Charles Onuselogu:** Data analysis, Writing – original draft preparation, review, and editing manuscript; **Jacob Olagbenro Popoola:** Writing manuscript, Supervision, Methodology, review of the manuscript; **Stephen Oluwanifise Oyejide:** Data analysis, review and editing of the manuscript.

## Declaration of Competing Interests

The authors declare that they have no known competing financial interests.

## Data Availability

Intra-Specific Genetic Variability Dataset on rbcL gene in Moringa oleifera LAM. (Moringaceae) in Nigeria (Original data) (genbank).
